# Vertebral osteomyelitis caused by *Campylobacter jejuni* in an immunocompetent patient

**DOI:** 10.1186/s13099-023-00589-2

**Published:** 2023-11-30

**Authors:** Karina Frahm Kirk, Jeppe Boel, Hans Linde Nielsen

**Affiliations:** 1 Department of Infectious Diseases, Aalborg University Hospital, Aalborg, 9000 Denmark; 2 Department of Clinical Medicine, Aalborg University, Aalborg, Denmark; 3 Department for Bacteria, Parasites & Fungi, Statens Serum Institut, Copenhagen, Denmark; 4 Department of Clinical Microbiology, Aalborg University Hospital, Hobrovej 18, Aalborg, 9000 Denmark

**Keywords:** *Campylobacter jejuni*, Vertebral osteomyelitis, Spondylodiscitis, Osteomyelitis, Bacteraemia, Gastroenteritis, Enteritis, Clindamycin, Ciprofloxacin resistance

## Abstract

**Background:**

*Campylobacter jejuni* is the leading cause of human bacterial gastroenteritis worldwide. However, systemic infection with *C. jejuni* is uncommon, and osteomyelitis caused by *C. jejuni* is extremely rare. Cultivation from spinal bone biopsies has not previously been reported in the literature.

**Case presentation:**

A 79-year-old immunocompetent male was admitted to the emergency department at Aalborg University Hospital in Denmark with lower back pain, fever and diarrhoea. A FecalSwab obtained upon admission was PCR-positive for *Campylobacter* spp, while an aerobic blood culture bottle was positive for *C. jejuni* (Time to detection: 70.4 h). A MRI of columna totalis showed osteomyelitis at L1/L2 with an epidural abscess from L1 to L2 with compression of the dura sack. The patient underwent spinal surgery with spondylodesis and decompression of L1/L2. The surgery was uncomplicated and the discus material was also culture positive for *C. jejuni*. The patient was treated with meropenem for a total duration of four weeks, followed by four weeks of oral treatment with clindamycin in tapered dosage. The patient recovered quickly following surgery and targeted antibiotic treatment with decreasing lumbar pain and biochemical response and was fully recovered at follow-up three months after end of treatment.

**Conclusions:**

While *C. jejuni* osteomyelitis is rare, it should still be suspected as a possible causative bacterial aetiology in patients with vertebral osteomyelitis, in particular when symptoms of diarrhoea is involved in the clinical presentation. Susceptibility testing is crucial due to emerging resistance, and targeted treatment strategies should rely upon such tests.

## Background

*Campylobacter* spp. are microaerophilic Gram-negative bacteria with a curved or spiral shape. The genus currently consists of 44 species validly published under the International Code of Nomenclature of Prokaryotes (ICNP) [1], of which at least 17 have been associated to human disease. The natural reservoir includes several warm-blooded animals, including wild birds, chicken and other poultry, cats, dogs, cows, as well as water sources in farm environments [2]. Campylobacteriosis is a human zoonosis. The main route of transmission being foodborne, via undercooked meat and meat products including poultry, and often follows a seasonal pattern with a peak midsummer [3]. The most common *Campylobacter* spp. to cause human disease are *C. jejuni* and *Campylobacter coli*, and the most common clinical presentation is self-limiting acute gastroenteritis, while systemic *Campylobacter* infections are rare and often associated to immunodeficiency [3]. Extra-intestinal manifestations caused by *Campylobacter* can include septic or reactive arthritis, skin and soft tissue infections, endocarditis and osteomyelitis, and such invasive infections are more commonly described in relation to *Campylobacter* spp. other than *C. jejuni* and *C. coli*, in particular *Campylobacter fetus* [2]. A previous study from Denmark reported an incidence of *Campylobacter* bacteraemia at 2.9 pr. million person-years, with highest risk in the elderly population with higher comorbidities [4]. In a recent five-year retrospective study from France, bacteraemia with *C. jejuni*/*coli* and *C. fetus* were the most common species identified, and 30-day mortality was reported to be as high as 11.7% among all patients with *Campylobacter* spp. bacteraemia [5]. Vertebral osteomyelitis is a rare disease, although worldwide incidence is generally increasing [6]. We here present a case of vertebral osteomyelitis with epidural abscess in an immunocompetent adult, caused by *C. jejuni*.

## Case presentation

A 79-year-old male was admitted to the emergency department at Aalborg University Hospital in Denmark in January 2023 with progressive lower back pain. On admittance, the patient was febrile and noted to have had diarrhoea for at least one week prior to hospitalisation, accompanied by mild complaints of diffuse abdominal pain. The patients’ medical history included prostate cancer, for which he was not yet actively treated, ischemic heart disease, atrial fibrillation, and previous stroke with negligible motor deficits, but the patient was immunocompetent. Vital parameters were stable, but the patient was febrile with a temperature of 38.1°C. Blood tests revealed an elevated C-reactive protein (CRP) level of 227 mg/l, but white blood cell (leucocyte) counts and procalcitonin were within the normal range (< 10 × 10^9/L and < 0.5 µg/L, respectively).

**Fig. 1:**
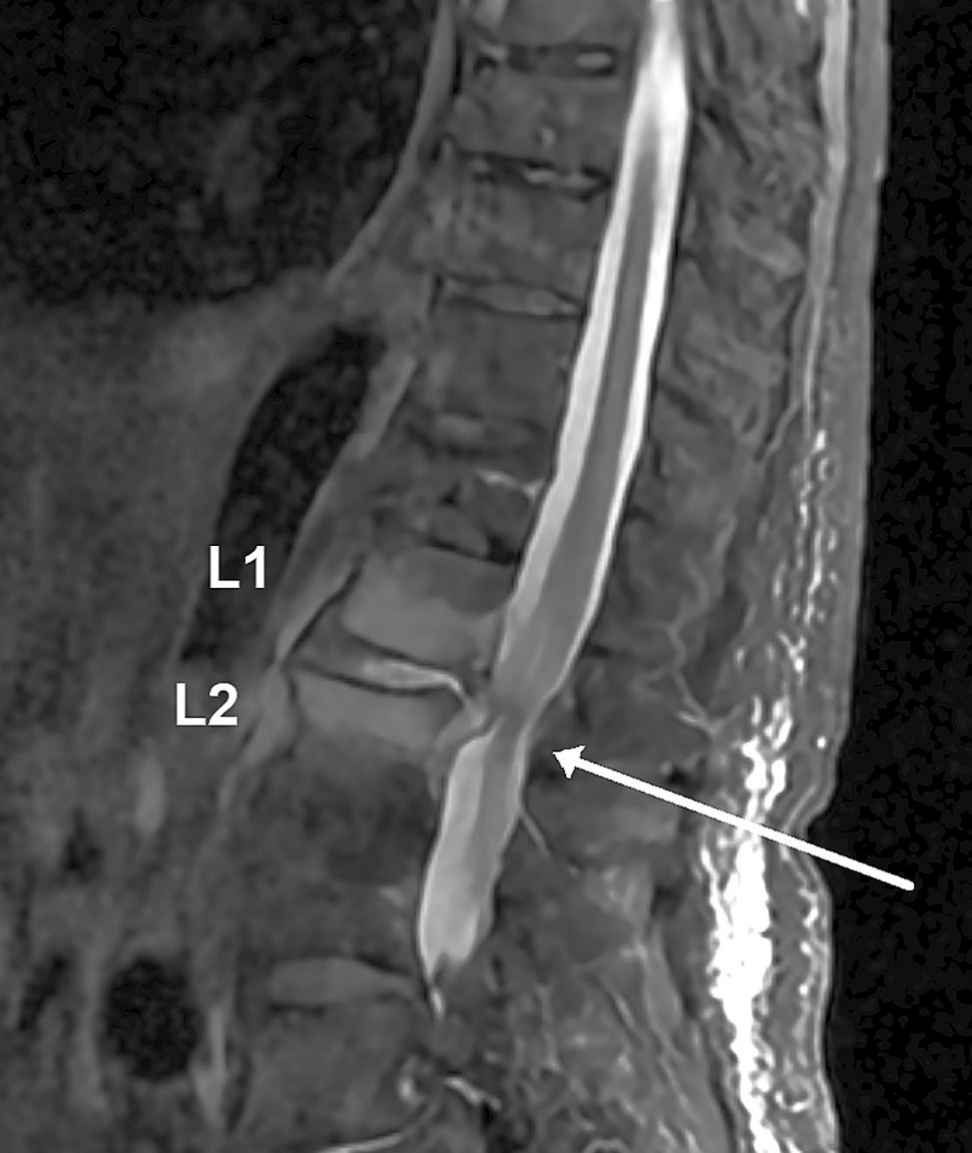
Sagittal STIR sequence of the lumbar spine showing hyperintensity signal of the L1/L2 intervertebral disc and the vertebral bodies of L1 and L2 with an epidural abscess, resulting in compression of the dura sack (white arrow)

A FecalSwab (Copan Italia, Brescia, Italy) was taken shortly after admittance, which was PCR-positive for *Campylobacter* spp. A CT scan of the thorax and abdomen showed terminal ileitis and signs of inflammatory discitis at L1/L2 suspect for osteomyelitis. A MRI of columna totalis confirmed suspicion of vertebral osteomyelitis with a hyperintensity signal of the L1/L2 intervertebral disc, and hyperintensity signal of both L1 and L2 vertebral bodies with an epidural abscess, resulting in compression of the dura sack, see Fig. 1. Three days after admission, a blood culture was positive with spiral-shaped, Gram-negative rods, later identified as *C. jejuni*.

Shortly following the positive blood culture, the patient underwent spinal surgery with spondylodesis and decompression of L1/L2 with donor autolog bonetransplantation in the disc space. The surgical procedure was uncomplicated, and cultivation from the spinal bone retrieved during surgery was also culture-positive for *C. jejuni*.

On admittance, the patient was empirically started on iv. piperacillin/tazobactam 4 g qad, and upon the blood culture results, iv. clarithromycin 500 mg bid was added. Following spinal surgery, treatment was targeted to iv. meropenem 1 g tid for 4 weeks, followed by 4 weeks treatment with clindamycin (2 weeks at dose 600 mg tid, tapered to 2 weeks dose of 300 mg tid). The patient recovered quickly following surgery, with decreasing lumbar pain and biochemical response, and was fully recovered at three months follow-up in the outpatient clinic. In an attempt to identify the source of *C. jejuni* infection, the patient reported having eaten undercooked turkey for Christmas dinner one week prior to hospitalization, but no other household members that ate the same meal had become ill.

### Investigations

A FecalSwab (COPAN ITALIA, Brescia, Italy) obtained 20 h upon submission was positive for *Campylobacter* spp. by use of the QIAstat-Dx® Gastrointestinal Panel (Qiagen, Hilden, Germany). A standard blood culture obtained upon submission (Two BD BACTEC™ Plus Aerobic medium and one BD BACTEC™ Lytic Anaerobic medium glass culture vials) was incubated in the BACTEC FX Top instrument (Becton Dickinson AB, Stockholm, Sweden). After three days of incubation there was growth in a single aerobic bottle (Time to detection: 70.4 h) of motile, Gram-negative, spiral-shaped rods. Sub-cultivation was performed on horse blood agar plates (SSI Diagnostica, Hillerød, Denmark) at 37 °C in a hydrogen-enriched microaerobic atmosphere (6% O_2_, 6% CO_2_, 6% H_2_, and 82% N_2_) and examined the day after (app. 18 h), with growth of non-haemolytic, greyish, smooth, colonies with a swarming growth. Colonies was catalase and oxidase positive and identified as *C. jejuni* by use of the matrix-assisted laser desorption ionization–time of flight (MALDI Biotyper 3.1, Bruker Daltonics Microflex LT, MBT 6903 MSP Library) with a score of 2.22.

Concurrently, one lumbar bone biopsy specimen (L1-L2) was sent to the microbiological laboratory. The bone specimen was cultured on standard solid culture media and incubated in environments containing 5% CO2, anaerobic conditions, and a hydrogen-enriched microaerobic atmosphere. Additionally, thioglycolate and serum broth were utilized. After 48 h, growth of *C. jejuni* was observed on all media, including blood agar, which was incubated in environments with 5% CO2, anaerobic conditions, and a hydrogen-enriched microaerobic atmosphere, respectively. Identification was confirmed through high MALDI scores. The bone specimen was also *Campylobacter* spp. positive by use the standard 16 S rRNA targeted next-generation sequencing platform performed at the reference laboratory at Statens Serum Institut (SSI) [7].

We conducted phenotypic antibiotic susceptibility tests (AST) of *C. jejuni* following the recommended method outlined in EU Decision 2013/652/EU [8], employing the Sensititre™ EU Surveillance Campylobacter EUCAMP3 AST Plate. For clindamycin, the minimum inhibitory concentration (MIC) was determined using a custom Sensititre™ plate with clindamycin concentration ranging from 0.25 mg/L to 8 mg/L. Following inoculation, the microplates were incubated at 41 °C under microaerobic conditions for 24 h. The results were interpreted according to EUCAST breakpoints (version 13.0) if available, interpreted based on epidemiological cut-off (ECOFF) values, as provided on the EUCAST website (www.eucast.org). The isolate exhibited low minimum inhibitory concentrations (MICs) and were interpreted as sensitive to chloramphenicol (MIC: ≤ 2 mg/L), gentamicin (MIC: ≤ 0.25 mg/L), tetracycline (MIC: ≤ 0.5 mg/L), and erythromycin (MIC: ≤ 1 mg/L), and resistant to ciprofloxacin (MIC: = 8 mg/L). The MIC values for clindamycin and ertapenem were = 0.5 mg/L and ≤ 0.12 mg/L), respectively.

Next, whole-genome sequencing was performed on the Illumina NextSeq instrument using the Nextera XT DNA Library Preparation Kit (Illumina, San Diego, USA) to produce paired-end reads. The raw reads and SKESA assembled genomes were submitted to and passed the SSI *in-house* QC pipeline (https://github.com/ssi-dk/bifrost). The assemblies were analyzed with the AMRinderPlus (https://github.com/ncbi/amr/wiki), Software version: 3.11.18, Database version: 2023-08-08.2, with settings coverage 0.5, identity 0.9, for the in-silico detection of acquired resistance genes and point mutations. Two resistance mechanisms were detected in the isolate, a point mutation in the *gyrA_T86I* gene conferring resistance towards ciprofloxacin, and an oxacillinase *blaOXA-461* family class D beta-lactamase. The WGS analysis further confirmed the isolate as *C. jejuni* and by use of the MLST 2.0 webtool (available at: http://www.genomicepidemiology.org) the isolated was assigned to sequence type ST-49.

## Discussion and conclusions

To our knowledge, this represents the first documented case of a patient with culture-positive *C. jejuni* vertebral osteomyelitis and epidural abscess in Denmark. It is also a reported case that necessitated combined surgical and pharmacological interventions for managing such an infection. Osteomyelitis is typically associated with risk factors such as diabetes, ischemic heart disease, cancer, immunosuppression, and male sex [9]. The underlying pathogenesis often involves hematogenous dissemination from another primary infection site; however, it can also result from direct inoculation due to trauma, surgery, or propagation from an adjacent soft tissue infection [10]. *Staphylococcus aureus* is the most commonly identified pathogen in cases of hematogenous spread, whereas haemolytic *Streptococcus* spp. are more frequently associated with skin or soft tissue infections [9]. In individuals hailing from endemic regions, considerations should extend to include tuberculosis and brucellosis [11, 12]. Enteric pathogens may be suspected in cases related to urogenital surgery, whereas a preceding history of gastrointestinal infection in osteomyelitis cases is less common. Although osteomyelitis caused by Salmonella has been previously reported in children with hemoglobinopathies, it has also been observed in immunocompetent adults [13]. Patients with osteomyelitis will often present with progressive diffuse back-pain, with fever being less frequently reported [14]. Remarkably, our patient exhibited symptoms of back pain, fever, and diarrhea, with the latter being relatively uncommon even in cases of osteomyelitis caused by *C. fetus* and Salmonella [13, 15].

Vertebral osteomyelitis in relation to Campylobacteriosis is a very rare complication, and in accordance with the recent findings from France, more often associated to *C. fetus* infection [5]. *Campylobacter fetus* is more commonly reported as a source of extraintestinal infections including bacteraemia and spondylodiscitis than *C. jejuni* [15–18]. Generally, extraintestinal infections and bacteraemia caused by *Campylobacter* spp. are associated with underlying conditions such as immunosuppression, diabetes, or cancer [4, 19]. Our patient had some medical comorbidities, but was otherwise immunocompetent. Symptoms on presentation included diarrhoea, and the first evidence of *Campylobacter* infection was therefore the FecalSwab, conducted quickly upon admission. While the QIAstat-Dx® Gastrointestinal Panel (Qiagen, Hilden, Germany) includes C. *jejuni, Campylobacter upsaliensis*, and *C. coli* it does not include *C. fetus*, and the suspicion of spondylodiscitis due to *C. jejuni* was most likely delayed due to the rarity of this condition. Initial treatment was therefore aimed at two or more possible infectious foci, with combination therapy.

Duration of treatment depends on the extent and complexity of infection and can consist of both surgical and antimicrobial therapy. Ideally, a multidisciplinary evaluation should precede treatment decisions [20]. Surgical intervention should be considered in cases featuring neurologic deficits, epidural or paravertebral abscesses, spinal instability, disease progression, or severe pain [20]. While studies regarding the optimal duration of antibiotic treatment are limited, current recommendations stipulate at least six weeks, with an initial two-week course of intravenous treatment. In cases of complicated osteomyelitis, treatment can extend up to 12 weeks [20–22]. Given our patient’s vertebral osteomyelitis complicated by an epidural abscess, treatment for eight weeks was deemed appropriate, especially considering the surgical debridement performed. In Denmark, the recommended empiric treatment of vertebral osteomyelitis with unknown aetiology, is iv. cefuroxime followed by oral moxifloxacin. For Enterobacterales, treatment is tailored based on resistance profiles, and is often 2nd or 3rd generation cephalosporines or a carbapenem. The literature concerning spondylodiscitis caused by *Campylobacter* spp. is sparse, and there are no established guidelines for specific antibiotic treatment strategies or durations for *Campylobacter* spondylodiscitis. Positive cultures from blood and spinal bone allowed for specific AST and targeted antibiotic treatment. The results of our AST were in coherence with overall resistance patterns for *C. jejuni* in Denmark, where resistance to ciprofloxacin is reported in almost half of domestically acquired human *C. jejuni* cases [23]. In previous case reports with *C. jejuni* spondylodiscitis, treatment has consisted of fluoroquinolones, macrolides or carbapenems, for a total duration of 5–10 weeks [24, 25]. While imipenem is commonly utilized in several countries, meropenem serves as the predominant carbapenem of choice in Denmark and was selected as the primary treatment for our patient in this case. Subsequently, oral clindamycin was administered as a stepdown therapy, leveraging its remarkable bone penetration capabilities, which position this antibiotic as an excellent candidate for managing bone infections. Nevertheless, its specific efficacy against *C. jejuni*-related bone infections remains unexplored. The *C. jejuni* strain exhibited a low MIC of = 0.5 mg/L towards clindamycin, and in vitro studies have consistently demonstrated clindamycin’s high effectiveness against various *C. jejuni* strains [26, 27]. Consequently, clindamycin may present a promising therapeutic alternative for the treatment of severe *C. jejuni* infections, including those affecting bone. Remarkably, the patient experienced no complications or reported side effects throughout both in-hospital and outpatient treatment, ultimately achieving a full recovery without adverse events, long-term sequelae, or the necessity for additional surgery.

In conclusion, while spondylodiscitis with *Campylobacter*, and in particular *C. jejuni* is rare, is should still be suspected in cases where *C. jejuni* is identified in any diagnostic samples, when back pain is a predominant symptom. There are no guidelines available regarding treatment, our patient was treated with antibiotics for a total duration of 8 weeks with a favourable outcome.

## Data Availability

Additional WGS-data is available on specific request to the corresponding Author.

## Abbreviations

AST: Antibiotic susceptibility testing
MIC: Minimum inhibitory concentration
MLST: Multi-locus sequence typing
ST: Sequence type
TTD: Time to detection
